# Over-expression of the constitutive AMPylase FIC-1(E274G) does not deplete cellular ATP pools in *C. elegans*

**DOI:** 10.17912/micropub.biology.000409

**Published:** 2021-06-25

**Authors:** Margaret Champion, Matthias C Truttmann

**Affiliations:** 1 University of Michigan Medical School, Department of Molecular & Integrative Physiology, Ann Arbor, MI

## Abstract

Protein AMPylation has emerged as a posttranslational protein modification regulating cellular proteostasis. AMPylation is conferred by Fic AMPylases, which catalyze the covalent attachment of AMP to target proteins at the expense of ATP. Over-expression of constitutive-active Fic AMPylases is toxic. Here, we test the hypothesis that excessive Fic AMPylase activity could deplete cellular ATP pools, leading to cell death. We find that increased/decreased Fic AMPylase activity only alters cellular ATP concentrations by approximately 15%. This suggests that hyper-AMPylation-mediated cell death is likely not the consequence of cellular ATP depletion.

**Figure 1. FIC-1 activity does not affect cellular ATP levels:**
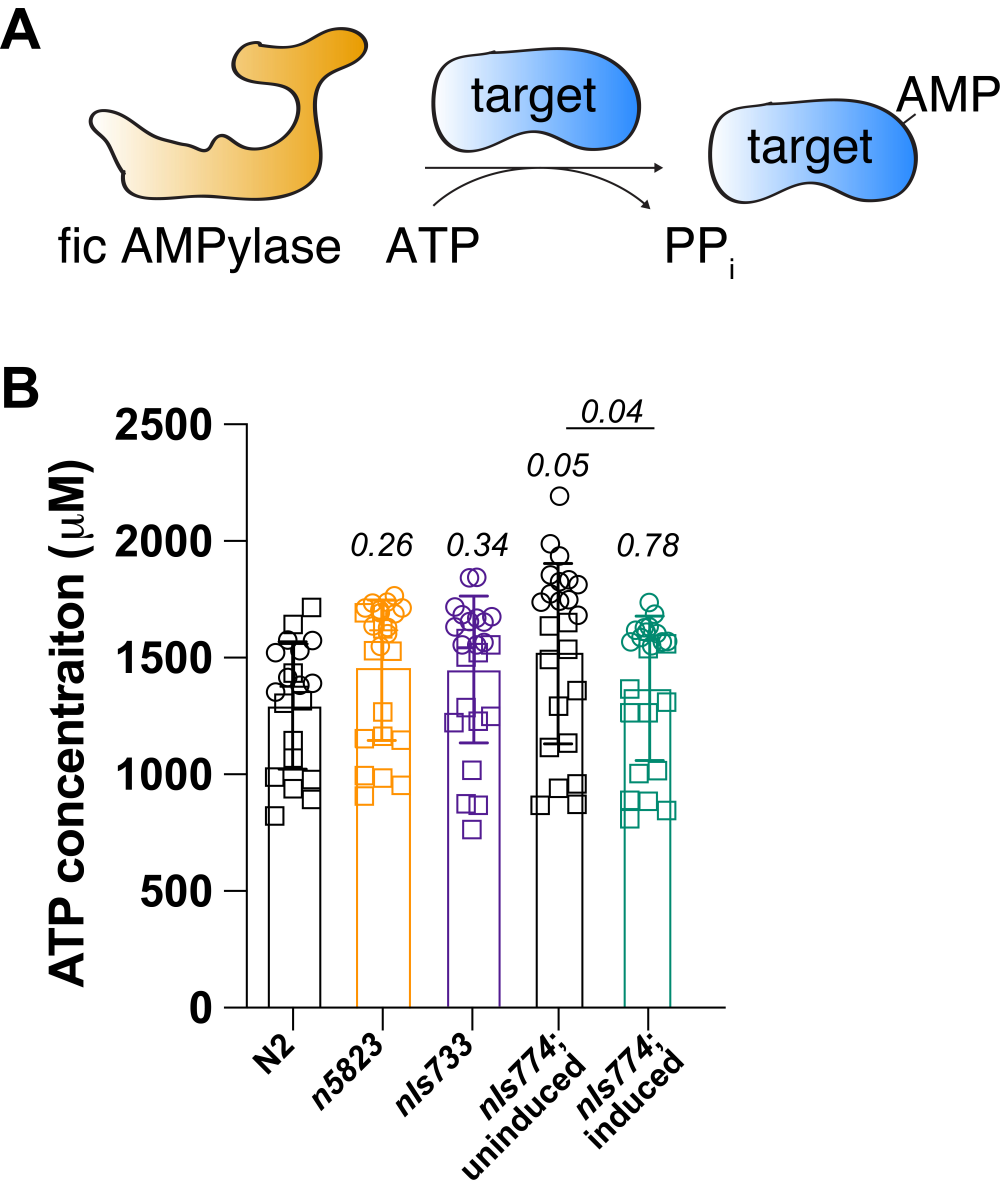
(**A**) Schematic of fic AMPylase-mediated target modification. (**B**) ATP concentration measurements in lysates of wild-type (N2), *fic-1* KO (*n5823*), FIC-1(E274G)-expressing (*nIs733*), and inducibly FIC-1(E274G) over-expressing (*nIs774*) worms. For (**B**): We used an ordinary one-way ANOVA test with multiple comparisons to calculate statistical significance. The comparison of uninduced and induced *nIs774* was done using the Mann-Whitney test. Circles and squares within samples highlight different biological replicates. Each circle/square depicts a single-well value (technical replica). The total dataset consists of two biological replicates, with 8 technical replicates each.

## Description

Fic domain-containing AMPylases (Fic AMPylases) are a family of evolutionarily conserved enzymes present in most metazoans. Fic AMPylases are bi-functional: as monomers, these enzymes catalyze the transfer of AMP – at the expense of an ATP molecule – to surface-exposed threonine and serine hydroxyl groups (target AMPylation) (Chatterjee and Truttmann, 2021; Perera *et al.*, 2019); upon dimerization and co-factor exchange, the same catalytic site supports AMP removal from modified proteins (target deAMPylation) (Casey *et al.*, 2018; Moehlman *et al.*, 2018; Preissler *et al.*, 2017a; Preissler *et al.*, 2017b; Veyron *et al.*, 2019). Work by many groups suggests that fic AMPylases modify proteins in the endoplasmic reticulum (ER) as well as in the cytoplasm, with a preference for the ER-resident chaperone BiP/Grp78 (**Fig. 1A**). The activity of fic AMPylases is tightly regulated. Fic AMPylase-mediated hyper-AMPylation, achieved by the over-expression of constitutive AMPylase mutants, is toxic and leads to cell death in human *in cellulo* models (Sanyal *et al.*, 2015), as well as *Saccharomyces cerevisiae* (Truttmann *et al.*, 2017), *Drosophila melanogaster* (Casey *et al.*, 2018) and *Caenorhabditis elegans* (Truttmann *et al.*, 2018) *in vivo* models. Hyper-AMPylation-mediated cell death involves caspase-dependent apoptotic signaling (Sanyal *et al.*, 2015). However, a detailed understanding as to why hyper-AMPylation is lethal remains elusive. One major hypothesis in the field is that hyper-AMPylation will deplete cellular ATP pools, leading to starvation-induced apoptosis.

Here, we tested this hypothesis by determining the intracellular ATP levels in *C. elegans* strains with increased/decreased fic AMPylase activity. *C. elegans* encodes for a single fic AMPylase, FIC-1. Mutating glutamine-274 to glycine renders FIC-1(E274G) into a constitutive-active AMPylase (Truttmann *et al.*, 2016). To determine whether FIC-1 activity affects cellular ATP levels, we measured intracellular ATP concentration of wild-type (N2), *fic-1* knock-out (*n5823*), and FIC-1(E274G)-expressing (*nIs733*) worms. Importantly, worms containing the *nIs733* transgene express the constitutive fic AMPylase FIC-1(E274G) under the control of the endogenous *fic-1* promoter, which leads to ubiquitous yet weak gene expression throughout the worm body (Truttmann *et al.*, 2016). In the same experiment, we also determined if the inducible over-expression of FIC-1(E274G) (*nIs774*) could affect cellular energy homeostasis. Since over-expression of FIC-1(E274G) is embryonically lethal in worms (Truttmann *et al.*, 2018), we induced FIC-1(E274G) over-expression in young adult worms for 4 hours before collecting and processing the worm lysates.

For each sample, we collected at least 1,000 animals per strain and replica and determined ATP concentrations in worm lysates using a luciferase-based assay optimized for *C. elegans* lysates (Kumsta *et al.*, 2011). We found that neither *fic-1* knock-out worms nor worms expressing low or high levels of the constitutive AMPylase FIC-1(E274G) show significant differences in ATP levels compared to N2 controls (**Fig. 1B**). Notably, the uninduced worm strain harboring the FIC-1(E274G) over-expression transgene (*nIs774*) showed a slight increase in intracellular ATP concentration compared to N2 (P=0.05). However, the mean difference in ATP concentration between N2 and uninduced *nIs774* worms was less than 15%, thus likely reflecting strain to strain variation in cellular ATP levels. Further, over-expression of FIC-1(E274G) reduced ATP levels compared to its uninduced control (P=0.04). However, the observed decrease in cellular ATP concentrations upon over-expression of FIC-1(E274G), while reproducible and statistically significant, is small, only leading to an approximately 12% reduction in cellular ATP content. Heat stress is known to reduce ATP levels in *C. elegans* (Yee *et al.*, 2014). Since the induction of FIC-1(E274G) over-expression requires a brief heat shock (30 minutes, 37C), the observed 12% decrease in ATP levels may reflect a combination of FIC-1(E274G)-dependent ATP consumption and heat stress-related decreases in cellular ATP concentrations.

Together, our experiments demonstrate that changes in FIC-1-dependent AMPylation activity are not a major determinant of cellular ATP levels. Rather, the effects are limited to fluctuations in ATP concentration of ± approximately 15%. Limited changes in cellular ATP levels in response to stress or aging are frequent and not necessarily linked to the immediate collapse of cellular functions (Huang *et al.*, 2010; Navarro and Boveris, 2007; Schutt *et al.*, 2012). Thus, it appears likely that the over-expression of constitutive-active fic AMPylase mutants is toxic/lethal to cells for reasons beyond increased cellular ATP consumption. Further experiments are required to determine the mechanistic basis of hyper-AMPylation-induced cell death.

## Methods

*C. elegans strains.* We used strains N2 (wild-type), MT22849 (*n5823* IV [*fic-1* KO]), MT23503 (*nIs733* [P*fic-1*::*fic-1*(E274G), P*myo-3*::mCherry]), and MT24419 (*nIs774* [P*hsp*–*16.2*::*fic-1*(E274G), P*myo-3*::mCherry]) for this study. All strains have previously been established and characterized (Truttmann *et al.*, 2016, Truttmann *et al.*, 2018). Worms were grown at 20 °C on NGM-agar plates spotted with *Escherichia coli* OP50. When needed, worms were synchronized by bleach prepping and thereafter grown at 20 °C on 6 cm NGM agar plates containing OP50 *E. coli*. To induce over-expression of FIC-1(E274G), day1 adult MT24419 worms were incubated for 30 minutes at 37 °C and immediately returned to 20 °C. Samples were collected 4 hours after induction. For all experiments, animals were collected by washing worms off plates with 1000 μl sterile M9, transferring the worms into 1.5 ml tubes, and centrifuging at 1,500 RPM for 60 seconds. Following three M9 washes, worm pellets were flash frozen in liquid nitrogen and stored at -80 °C until sample preparation.

*Sample Preparation.* Worm pellets of day1 adult worms were thawed on ice, resuspended in 100 μl sample buffer and lysed using a Qiagen TissueLyserII (30 Hz, 5 minutes). Protein concentration in each lysate was determined via Bradford assay. Samples were boiled in guanidinium isothiocyanate (GITC) at 100°C for 10 minutes and then centrifuged at 13,000 RPM at 4 °C for 30 minutes. The ATP-containing supernatant was thereafter transferred into a fresh 1.5 ml tube. Lysate protein concentration was normalized to the lowest concentration by diluting supernatant in sample buffer. Finally, supernatants were diluted in 1:200 in sample buffer in preparation for ATP measurements.

*ATP Measurements*. We used an ATP-dependent luciferase/luminescence assay to determine ATP concentrations. For this,20 μl of diluted supernatants were added to the wells of a flat 96-well opaque Costar plate and stored on ice. To construct the standard curve, we further added20 μl of 0, 0.5, 1, 2, 5, 7.5, 10, and 100 μM ATP diluted in sample buffer to separate wells of each 96 well plate. Each well was then supplemented with 90 μl of detection buffer. This standard curve was then used to determine the absolute ATP concentration in each sample. The luciferase reaction was quantified using an Omega BMG Fluorstar plate reader. Luminescence was read at around 2000-3000 gain in 45 seconds intervals over 7 cycles, with plates being agitated.

## Reagents

**Sample buffer:** 40 mM HEPES, 4 mM MgSO_4_, pH 7.8.

**Detection buffer:** 1X luciferase buffer, 0.1 mg/ml bovine serum albumin, 1 mM dithiothreitol, 100 μM luciferin in 25 mM glycylglycine pH7.8, 25 nM luciferase. Luciferase should be kept away from any light source and added immediately before measurement.

**20X Luciferase buffer:** 500 mM Tricine buffer pH 7.8, 100 mM MgSO_4_, 2 mM EDTA, 2 mM sodium azide.
